# SPARC, FOXP3, CD8 and CD45 Correlation with Disease Recurrence and Long-Term Disease-Free Survival in Colorectal Cancer

**DOI:** 10.1371/journal.pone.0022047

**Published:** 2011-07-26

**Authors:** Angela Chew, Paul Salama, Anneli Robbshaw, Borut Klopcic, Nikolajs Zeps, Cameron Platell, Ian C. Lawrance

**Affiliations:** 1 Centre for Inflammatory Bowel Diseases, Fremantle Hospital, Fremantle, Western Australia, Australia; 2 School of Medicine and Pharmacology, University of Western Australia, Fremantle, Western Australia, Australia; 3 School of Surgery, University of Western Australia, Nedlands, Western Australia, Australia; 4 Radiation Oncology, Sir Charles Gairdner Hospital, Nedlands, Western Australia, Australia; 5 St John of God Pathology, Subiaco, Western Australia, Australia; 6 St John of God Colorectal Service, Subiaco, Western Australia, Australia; The University of Hong Kong, China

## Abstract

**Background:**

SPARC is a matricellular protein involved in tissue remodelling, cell migration and angiogenesis, while forkhead box P3 (FOXP3) protein functions as a transcription factor involved in immune cell regulation. Both SPARC and FOXP3 can play an anti-tumorigenic role in cancer progression. The aim was to determine if SPARC, FOXP3, CD8 and CD45RO expression levels are associated with colorectal cancer (CRC) stage, disease outcome and long-term cancer-specific survival (CSS) in stage II and III CRC.

**Methods and Findings:**

SPARC expression was initially assessed in 120 paired normal and stage I-IV CRCs. Subsequently, approximately 1000 paired patient samples of stage II or III CRCs in tissue microarrays were stained for SPARC, FOXP3, CD8 or CD45RO. Proportional hazards modelling assessed correlations between these markers and clinicopathological data, including disease outcome and cancer specific survival (CSS). Both SPARC and FOXP3 expression were significantly greater in CRC than normal colon (p<0.0001). High SPARC expression correlated with good disease outcome (≥60 mths without disease recurrence, p = 0.0039) and better long-term CSS in stage II CRC (<0.0001). In stage III CRC, high SPARC expression correlated with better long-term CSS (p<0.0001) and less adjuvant chemotherapy use (p = 0.01). High FOXP3 correlated with a good disease outcome, better long-term CSS and less adjuvant chemotherapy use in stage II (p<0.0037, <0.0001 and p = 0.04 respectively), but not in stage III CRC. High CD8 and CD45RO expression correlated with better disease outcome in stage II CRC, and better CSS, but the differences were not as marked as for SPARC and FOXP3.

**Conclusions:**

These data suggest that high SPARC and FOXP3 are associated with better disease outcome in stage II CRC and may be prognostic indicators of CSS. Further assessment of whether these markers predict patients at high risk of recurrence with stage II CRC and functional studies of these effects are underway

## Introduction

Colorectal cancer (CRC) is highly prevalent in western populations and one of the leading causes of cancer morbidity and mortality worldwide[1], [2]. Excluding non-melanoma skin cancer, CRC is the most common cancer in Australia and the second most common cause of death due to malignant disease[3], [4]. The American Joint Committee on Cancer (AJCC) developed a commonly used staging system to determine patient prognosis. There is, however, overlap in the 5-year survival rates between stages II and III CRC[5], [6]. Stage III cancers have demonstrated lymphatic spread whilst stage II cancers have tumours localised to the intestinal mucosa. Some of the stage II tumours, however, may also have micrometastases that go undetected. In view of this, consideration of the biological characteristics of the tumour, through the identification of biomarkers that predict disease extent, may provide a more accurate means of correlating cancer stage with disease outcome, and allow application of the most appropriate treatments for individual patients.

Tumour infiltrating T lymphocytes that express CD8 and CD45RO have been shown to correlate with better CRC outcomes[7], [8], [9], [10]. The presence of a high density of CD8^+^ T cells is also associated with the absence of tumour invasion, an earlier disease stage and improved patient survival[11], [12]. High CD45RO^+^ T cell densities in lymph node metastasis and tumours of CRC also associate with less tumour invasiveness, lower disease stage and a better patient survival[9], [12]. Two further potential biomarkers of interest in the pathogenesis and prognosis of CRC are Secreted Protein Acidic and Rich in Cysteine (SPARC) and forkhead box P3 (FOXP3) as they are also both highly expressed in malignant diseases and have been demonstrated to significantly correlate with disease outcome.

SPARC is a matricellular glycoprotein that is involved in tissue remodelling, wound repair, cell migration and angiogenesis[13], [14], [15], [16]. It is expressed in numerous tissues, especially in damaged tissues where extracellular matrix (ECM) remodelling is necessary in response to injury[17]. High SPARC expression has been associated with enhanced tumour growth, metastasis and poor disease prognosis in various malignancies including melanoma, breast and oesophageal cancers[6], [18], [19]. SPARC has also been implicated in colonic polyp development and CRC tumour progression through the regulation of cycloxygenase-2 (COX-2)[20] and transforming growth factor beta-1 (TGFβ-1)[21]. These are involved in regulation, and breakdown, of the ECM surrounding tumour cells. In ovarian cancers, however, high SPARC expression levels are associated with a good prognosis[22]. This correlation is a result of SPARC's ability to down-regulate macrophage recruitment to the tumour site and the production and activity of interleukin (IL)-6, prostanoids and matrix metalloproteinases to decrease the associated inflammation[23].

FOXP3 is also a biological marker of interest and is the key transcription factor controlling regulatory T cell (Treg) development and function. Tregs suppress the activation of the immune system, thereby maintaining immune system homeostasis and tolerance to self-antigens. In doing so, however, these cells can suppress the anti-tumour immunological response by reducing cytotoxic T cell activity by direct cell-to-cell contact, or through the release of cytokines[24]. A direct link between the presence of Tregs and the progression of ovarian cancer has been demonstrated, where tumour FOXP3+ Tregs suppressed tumour specific immunity and contributed to reduced survival[25]. In breast cancer, an increase in the Treg population, both in peripheral blood and tumour tissue, was also reported and a recent study demonstrated a correlation between intratumoural infiltration of FOXP3+ Tregs in breast cancer and the risk of late relapse[26]. Given the central contribution of FOXP3 to Treg function, the expression of FOXP3 by tumour cells may represent a novel mechanism by which cancers suppress the immune system to escape destruction.

In this study we investigated SPARC and FOXP3 in stage II and III CRC tissue and compare their prognostic value against the known CRC prognostic markers CD8 and CD45RO that are expressed by tumour infiltrating T lymphocytes. These have been shown to correlate with a better CRC disease outcome[7], [8], [9], [10]. The presence of a high density of CD8^+^ T cells is associated with the absence of tumour invasion, an earlier disease stage and improved patient survival[11], [12].

The aims of this study were to determine if SPARC and FOXP3 expression levels are associated with CRC stage, disease outcome and long-term cancer-specific survival in stage II and III CRC and to compared the prognostic value of these two markers against the known prognostic CRC markers CD8 and CD45RO.

## Materials and Methods

### Ethics statement

Ethical approval for this research was obtained from the Human Research Ethics Committee of the Southern Metropolitan Area Health Services and from the Sir Charles Gairdner Hospital Human Research Ethics Committee. All patients providing tissue for the tissue microarray signed a consent form prior to surgical removal of the intestinal cancer to allow for this research to be undertaken.

### Patient details

Demographic information was collected retrospectively on all patients from pathology records, and information on disease-specific survival was obtained from the Cancer Registry of Western Australia. All patients had a minimum follow-up of 60 months or until death. Information on the use of adjuvant chemotherapy with fluorouracil/leucovorin-based regimens following surgery was obtained from hospital medical records. Patients were considered to have had a good disease outcome if there was no disease recurrence within 60 months of diagnosis, while patients who had disease recurrence within 60 months of diagnosis, or had died from their disease, were considered to have had a poor disease outcome.

### Initial SPARC assessment

SPARC levels were initially assessed in histological sections of AJCC stage I to IV CRC tissue to determine if there was an association with CRC and in which cells SPARC was expressed. This included samples (n = 120) collected from a 464 patient cohort diagnosed with CRC between 1996 and 2002.

### Tissue microarray (TMA)

Construction of the TMAs used in this study has previously been described[27]. The TMAs consisted of approximately 1000 cases each of stage II and III CRC archival tissue samples from patients who were diagnosed with CRC during the period of 1990 to 1999 at the Sir Charles Gairdner Hospital. Each case consisted of two 1mm diameter tumour tissue cores and an additional 1mm diameter core taken from histologically normal colon mucosa.

Assessment of SPARC expression was undertaken on 233 patients with AJCC stage II and 125 AJCC stage III CRCs. T cell density analysis of FOXP3, CD8 and CD45RO was derived from a larger patient cohort of the same archived AJCC stage II and III CRC samples that has previously been described[28].

Four micrometer thick formalin-fixed paraffin embedded sections from the TMAs had high temperature antigen retrieval with 1 mM EDTA (pH 8) and 10 mM citrate buffer (pH 6). Non-specific staining was blocked. Primary antibodies anti-SPARC (1:200; Hematologic Technologies Inc, Vermont, USA), anti-CD8 (clone C8/144B, ready to use; DakoCytomation, Heverlee, Belgium), CD45RO (clone UCHL1, ready to use; DakoCytomation) and anti-FOXP3 (1∶100; Abcam, Cambridge, MA, USA) were used with an overnight incubation at 4°C. After washing, the sections were incubated with 1∶2 diluted biotinylated link universal antibody and streptadvidin-HRP solutions (Universal LSAB+ kit, DakoCytomation Carpinteria, CA) for 30 minutes each. Colour was developed by incubating in 3,3-diaminobenzidine (DAB) solution (Substrate Chromogen System, DakoCytomation, Carpinteria, CA) and sections were counterstained with Mayers haemotoxylin and coverslipped with Depex. Staining without primary antibody was used as a negative control. Pictures were taken using a digital camera (Nikon DS-L1) in bright field on an inverted microscope (Nikon TE2000-U). Tissue microarrays were scanned using the Aperio ScanScope XT Digital Slide Scanner (Aperio Technologies, CA) located at the Australian Microscopy & Microanalysis Research Facility at the Centre for Microscopy, Characterisation & Analysis, The University of Western Australia

### Assessment of SPARC expression

**Figure 1:**
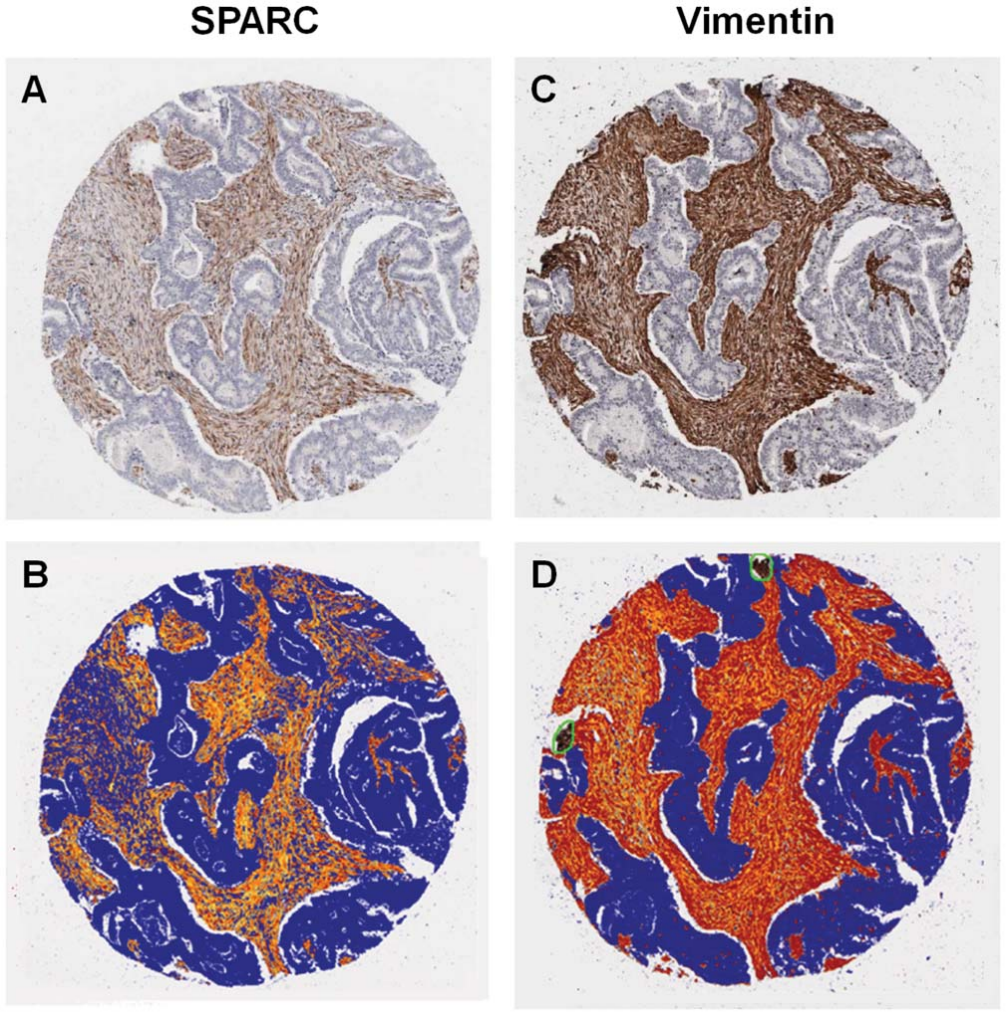
Tissue microarray analysis. SPARC and vimentin staining from a single patient. Images demonstrate serial DAB-stained tissue sections for both (**A**) SPARC and (**C**) vimentin and computer analysed images of the respective DAB staining (**B and C**). Blue represents DAB-negative pixels (dark areas), orange DAB-positive pixels and red strongly DAB-positive pixels (light areas). SPARC expression was selectively measured only in the regions of the tissue that were also vimentin-positive.

The role of SPARC in the pathogenesis of different tumours is highly contextual. In both CRC, and normal colonic tissue, SPARC was noted to be almost exclusively expressed by vimentin-positive stromal mesenchymal cells. Its expression was not observed in the malignant cells and was seen in only a very small number of normal epithelial cells. SPARC expression within malignant and control tissue, therefore, needed to be controlled for the amount of stromal tissue present. Serial tissue sections were taken and stained for SPARC and the specific mesenchymal cell marker, vimentin (anti-vimentin; 1∶30; Novocastra Laboratories Ltd). The vimentin-positive cells were morphologically identified by an independent histopathologist as stromal mesenchymal cells. SPARC positive areas were determined using a positive pixel count algorithm (ImageScope v8.0, Aperio Technologies, CA), which measures the staining intensity of the positive, strong-positive and negative pixels ***(*** ***Figure 1*** ***)***. SPARC expression was only measured in regions containing vimentin-positive cells and the ratio of SPARC to vimentin staining for each TMA core was used to control for the stromal tissue content within each sample. All SPARC results are presented as a ratio of SPARC to vimentin.

### Assessment of FOXP3, CD8 and CD45RO expression

The measurement of T-cell density has been previously reported[28] and used image analysis software (Imagescope v8.0, Aperio) to evaluate the number of FOXP, CD8 and CD45RO positive cells (cells per square millimetre). Individual cores were examined and annotated to ensure that only normal colonic epithelium or viable tumour tissue was included in the area of analysis.

### Statistical analysis

Results were expressed as mean ± SEM. Student's unpaired T-test and Kruskal-Wallis analysis with Dunn's post tests were used to compare SPARC expression and T-cell density between the different groups, and a P value of <0.05 was considered statistically significant. SPARC expression and T-cell densities were classified as *high* or *low* in relation to the median value as shown in Salama *et al*, 2008[28]. All statistical analyses and graphs were created using Graphpad Prism® 4.0 software package for Windows PC (Graphpad Software, San Diego, CA, USA). The Kaplan-Meier product limit estimate of survival was used to calculate long-term cancer-specific survival. The event variable was death from CRC (as defined by coding on the patient's death certificate). All survival times were calculated from the date of histological diagnosis of CRC until either an event occurred or they remained alive at 1^st^ March 2008.

## Results

### Stromal SPARC expression in normal colonic tissue vs. stage I to IV CRC

**Figure 2:**
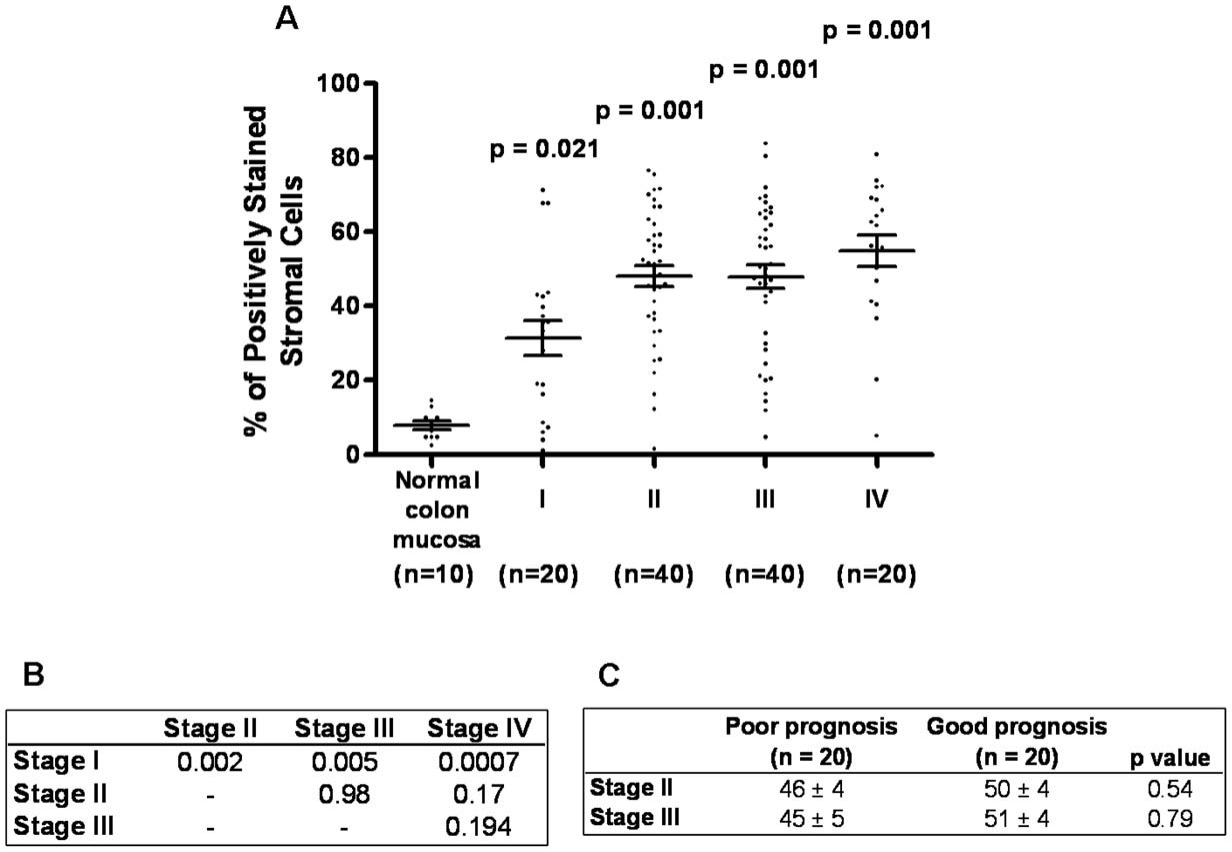
Correlation between SPARC expression and AJCC disease staging. (**A**) Percentage of positively stained cells in AJCC stage I, II, III and IV CRCs compared to normal colonic tissue (Con). SPARC expression was significantly greater in CRC compared to control. There was a significantly greater number of SPARC positive cells in stage II, III and IV CRC compared to stage I (p = 0.092, p = 0.005 and p = 0.0007 respectively). Data presented as a scatter plot with the mean ± SEM. (**B**) P values comparing the percentage of SPARC positive cells in stage I, II, III and IV CRC. (**C**) P values comparing SPARC expression in stage II to III good and poor outcome CRC. No differences in SPARC levels between good and poor stage II CRC or good and poor stage III CRC. Data expressed as a percentage of positively stained cells ± SEM.

SPARC levels were examined in 120 paired normal colonic and AJCC stages I to IV CRC. SPARC expression was significantly greater in all cancers compared to the corresponding normal colonic tissue ***(*** ***Figure 2A*** ***).*** It was also significantly greater in CRC stages II to IV compared to stage I, however, there were no differences between stages II to IV ***(*** ***Figure 2B*** ***)***. When patients with stage II and III CRC were divided into those with a good outcome (no disease recurrence within 5 years of diagnosis) and a poor outcome (disease recurrence within 5 years of diagnosis or death from disease), patients with a good disease outcome in both stage II and III had higher SPARC expression in the primary tumour, but these findings were not significantly different ***(*** ***Figure 2C*** ***).***

### Stromal SPARC expression in stage II and III CRC

**Figure 3:**
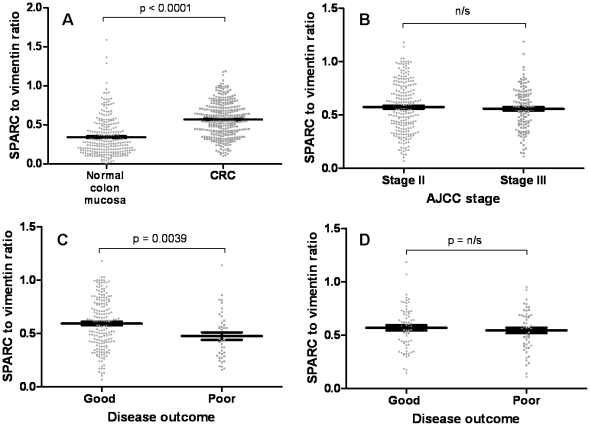
SPARC expression in stage II and III CRC using TMAs. (**A**) The SPARC to vimentin ratio was significantly greater in CRC tissue compared to normal colonic tissue. (**B**) No difference in SPARC expression was observed between stage II and III CRC. (**C**) SPARC was significantly greater in patients with a good disease outcome in stage II CRC, but not in (**D**) stage III CRC. Each dot represents one patient and the bars represent the median value ± SEM.

TMAs were employed to measure SPARC in a cohort of 358 stage II or III CRC patients. SPARC expression was significantly greater in all cancer (stage II and III combined) compared to normal colonic tissue (p<0.0001; ***Figure 3A***), with no statistical differences detected in SPARC expression between patients with stage II and III disease (***Figure 3B***). When patients were further divided into stage II and III disease with either a good or a poor outcome, SPARC expression was significantly greater in patients with a good disease outcome in stage II CRC (p = 0.0039; ***Figure 3C***), however, no statistical difference was observed between the groups in stage III CRC (***Figure 3D***).

### T-cell FOXP3, CD8 and CD45RO expression in CRC

**Figure 4:**
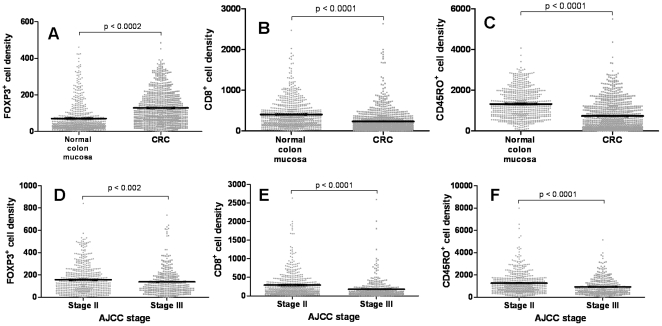
FOXP3, CD8 and CD45RO expression in CRC and their effects on cancer stage and disease outcome. (**A**) FOXP3 expression was significantly greater in CRC tissue compared to normal colonic tissue, whilst (**B**) CD8 and (**C**) CD45RO expression levels were significantly greater in normal colonic tissue compared to CRC tissue. When looking at T cell density and disease stage (**D** – FOXP3; **E** – CD8 and **F** – CD45RO), the expression of all three markers were significantly greater in stage II CRC compared to stage III CRC. Each dot represents one patient and the bars represent the median value ± SEM.

**Figure 5:**
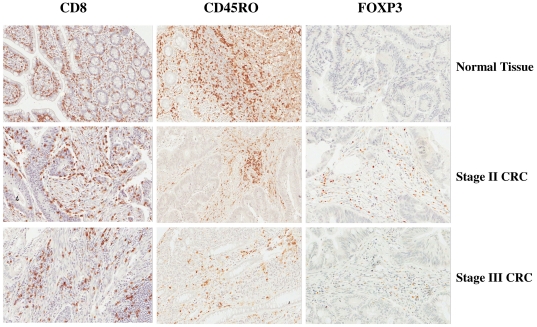
Representative images of CD8, CD45RO and FOXP3 staining in normal colonic tissue, stage II and III CRC. Images demonstrate DAB-stained tissue sections for CD8 positive cells, CD45RO-positive cells and FOXP3 positive cells. Images were taken at 20X scanning magnification.

**Figure 6:**
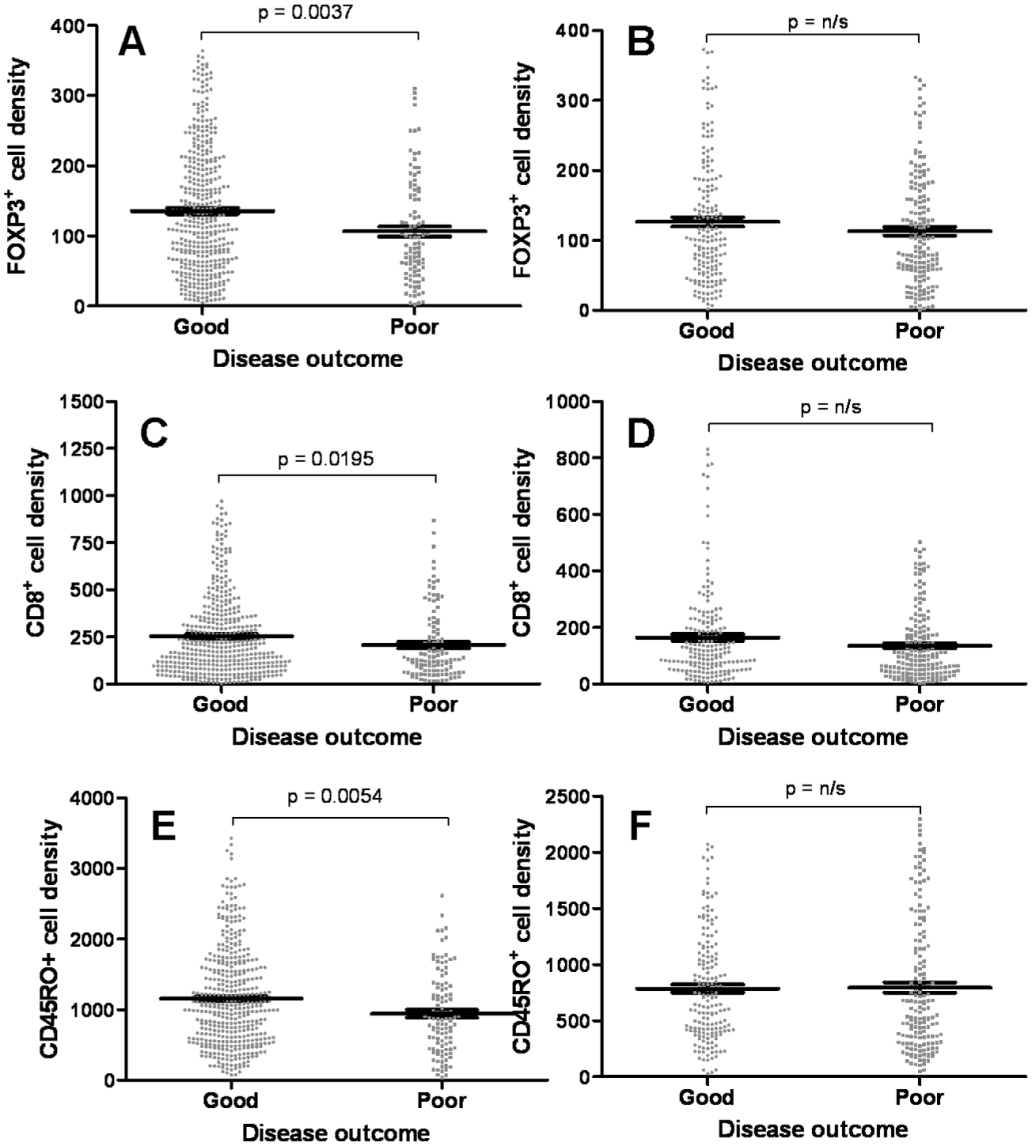
T cell density and disease outcome. When dividing the patients into good and poor disease outcome, FOXP3 expression was significantly greater in patients with a good disease outcome in (**A**) stage II CRC but not in (**B**) stage III CRC. Similarly, CD8 was also significantly greater in patients with a good disease outcome in (**C**) stage II CRC but not in (**D**) stage III, as with CD45RO in (**E**) stage II and (**F**) stage III. Each dot represents one patient and the bars represent the median value ± SE.

Examining T cell density using TMAs of a 967 cohort, FOXP3 was significantly higher in CRC tissue compared to normal colonic mucosa (p<0.002, ***Figure 4A*** ***and*** ***Figure 5***). In contrast, CD8 and CD45RO expression levels were significantly lower in normal colonic mucosa (p<0.0001, ***Figure 4B and C***) consistent with previously findings. When dividing the samples into stages II or III, FOXP3 was significantly greater in stage II compared to stage III (p<0.002, ***Figure 4D***). Similarly, CD8 and CD45RO expression levels were significantly greater in patients with stage II CRC compared to stage III (p<0.0001 for both, ***Figure 4E and F***). When comparing these markers with disease outcome, FOXP3 was significantly greater in patients with a good compared to poor outcome in stage II CRC (p = 0.0037; ***Figure 6A***), whilst no statistical differences were observed in stage III CRC (***Figure 6B***). These results were similar for CD8 and CD45RO (p = 0.0195 and 0.0054 respectively, ***Figure 6C, D, E and F***).

### Cancer-specific survival

**Figure 7:**
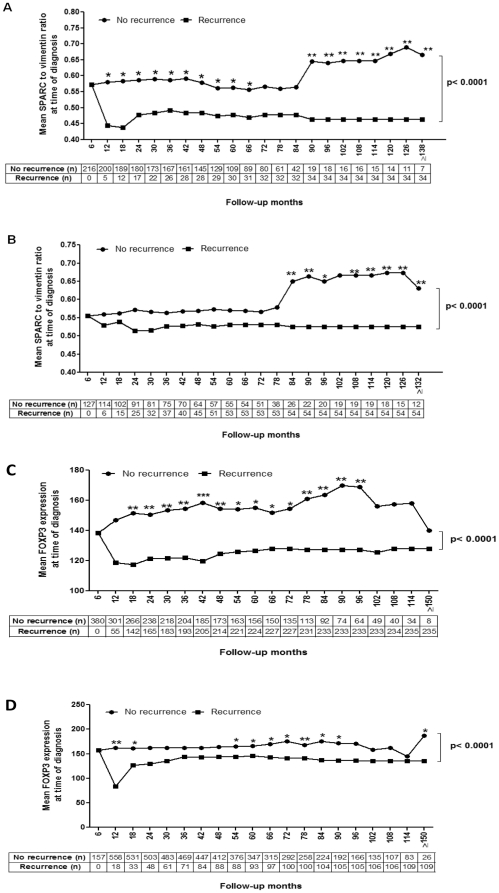
Cancer-specific survival. The mean SPARC and FOXP3 expression in the primary tumour was measured and correlated with disease recurrence. The number of patients with or without disease recurrence at each time point is displayed below each graph. (**A**) ***SPARC expression in stage II CRC*** *.* Patients without disease recurrence had significantly higher SPARC levels at all time point up to 60 months and for most points up to 138 months compared to those patients who suffered a disease recurrence. The overall SPARC expression was significantly different between the two groups (p<0.0001). (**B**) ***SPARC expression in stage III CRC*** *.* SPARC expression was significantly higher in patients without disease recurrence from 84 months and beyond. SPARC was also significantly different between the two groups (p<0.0001). (**C**) ***FOXP3 expression in stage II CRC*** **.** Higher FOXP3 levels was also significantly associated with less disease recurrence as with (**D**) ***FOXP3 expression in stage III CRC*** **,** however, the differences were not as marked as in stage II CRC. FOXP3 expression was significantly different between the two groups in both stage II and III (p<0.0001). Each data point represented the mean SPARC or FOXP3 expression level at each time point. (*) p<0.05, (**) p<0.01.

Patients were followed a minimum of 60 months or until death. The median follow-up time was 60.6 months for patients with AJCC stage II disease and 41.3 months for patients with stage III disease. The mean SPARC and FOXP3 expression in the primary tumours of patients with and without disease recurrence at each time point were measured to determine if the levels correlated with disease recurrence over time. Stage II CRC patients without disease recurrence, had significantly higher SPARC levels in their primary tumours at all time points up to 60 months, and for most points up to 138 months, compared to those patients suffering a disease recurrence (***Figure 7A***). In stage III CRC, SPARC levels were significantly higher in patients without disease recurrence 84 months from diagnosis and onwards (***Figure 7B***). This association between high SPARC expression and low disease recurrence was reinforced by univariate analysis demonstrating that the difference between the two groups (with, and without, disease recurrence) was significantly different in both stage II and III (p<0.0001; ***Figure 7A and B***).

Higher FOXP3 levels were also associated with less disease recurrence in stage II CRC (***Figure 7C***), and although the FOXP3 expression levels in the primary tumours were different between patients with and without disease recurrence in stage III as various time points (***Figure 7D***), these differences were not as marked as in stage II disease. As with SPARC, the overall FOXP3 expression was significantly different between the patients with, and without, disease recurrence in both stage II and III (p<0.0001: ***Figure 7C and D***).

### Overall survival

**Figure 8:**
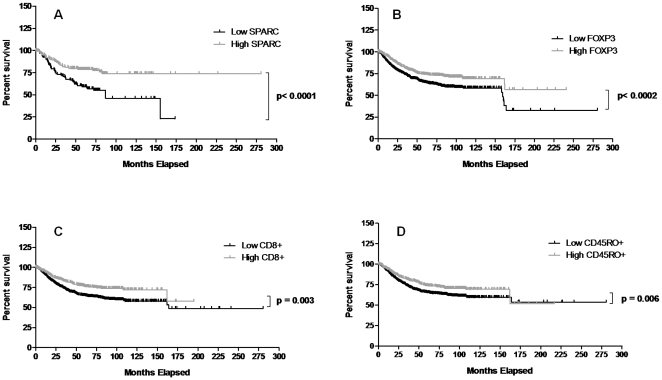
Overall survival. Kaplan-Meier survival curves were derived to show high and low marker levels in relation to survival. (**A**) High SPARC levels significantly correlated with better long-term survival. (**B**) High FOXP3 levels also significantly correlated with better long-term survival. Although high (**C**) CD8 and (**D**) CD45RO expression levels were associated with better long-term survival, the separation and significance between the curves were not as great.

By the end of the study period, 27% of patients had died from CRC and 22% had died from other causes. To assess overall patient survival, Kaplan-Meier survival curves were derived to show high and low marker levels in relation to the median SPARC value, and their association with survival. High SPARC and FOXP3 levels both significantly correlated with a better long-term survival (p<0.0001 and 0.0002 respectively; ***Figure 8A and B***). High CD8 and CD45RO levels also correlated with a better long-term survival, but compared to SPARC and FOXP3, the separation between the curves and the significance levels were not as great (***Figure 8C and D***). A univariate survival analysis was used to determine the prognostic significance of each of the four markers. ***Table 1A*** shows that high SPARC, FOXP3, CD8 and CD45RO expression in CRC tissue all independently predicted survival. Combining all four markers in a multivariate analysis (***Table 1B***) demonstrated, that while each of these markers had significant prognostic value on their own, used together they did not improve the prediction of survival. The combination of SPARC and FOXP3, or SPARC and CD8, however, significantly predicted survival better than using all four markers in concert, or the combination of FOXP3, CD8 and CD45RO (***Table 1B***). None of these combinations, however, were better at predicting survival than SPARC used alone.

**Table 1:** (A) Univariate survival analysis for SPARC, FOXP3, CD8 and CD45RO in CRC tissue from patients with stage II and III CRC.

A. Univariate survival analysis for SPARC, FOXP3, CD8 and CD45RO in CRC tissue from patients with Stage II and III CRC			
Marker	OR	95% CI	p value
SPARC	0.96	0.94 to 0.98	0.0001
FOXP3	0.86	0.74 to 1.0	0.05
CD8	0.83	0.72 to 0.96	0.12
CD45RO	0.84	0.72 to 0.97	0.017
NB: Univariate analysis, COX proportional hazards regression.			

B. Multivariate analysis for significant prognostic indicators in CRC tissue from patients with Stage II and III CRC			
Marker	OR	95% CI	p value
SPARC	0.96	0.94 to 0.98	0.0001
FOXP3	0.90	0.76 to 1.0	0.219
CD8	0.85	0.71 to 1.0	0.0.58
CD45RO	1.00	0.84 to 1.2	0.916
**Combination I**			
SPARC	0.96	0.94 to 0.98	0.0001
FOXP3	0.85	0.74 to 0.99	0.0034
Overall significance			0.0001
**Combination II**			
SPARC	0.96	0.94 to 0.98	0.0001
CD8	0.82	0.71 to 0.95	0.01
Overall significance			0.0001
**Combination III**			
FOXP3	0.92	0.77 to 1.1	0.31
CD8	0.87	0.73 to 1.0	0.10
CD45RO	0.96	0.80 to 1.1	0.65
Overall significance			0.065

High expression levels of all markers significantly predict patient survival. **(B)** **Multivariate analysis for significant prognostic indicators in CRC tissue from patients with stage II and III CRC**. All four markers combined did not improve the predictive value over individual markers. The combination of SPARC and FOXP3, or SPARC and CD8, predicted survival better than all four markers together, or the combination of FOXP3, CD8 and CD45RO, but no combination improved on the predictive sensitivity of SPARC used alone.

NB: COX proportional hazards regression model. Abbreviations: CRC – colorectal cancer, OR – odds ration, 95% CI– 95% confidence interval.

### Correlation of CRC pathology with T-cell density and SPARC expression

The pathological features of CRC, T-cell markers and SPARC expression are listed in ***Table 2***. The density of CD8^+^, CD45RO^+^ and FOXP3^+^ cells in tumour tissue inversely correlated with AJCC stage (consistent with the TMA data; ***Figure 4A***), whilst no significant correlation between SPARC expression and AJCC was observed (also consistent with the TMA data ***Figure 3B***). CD8^+^ and CD45RO^+^ expression inversely correlated to perineural invasion, and directly correlated with lymphocytic response and microsatellite instability.

**Table 2:** Correlation of CRC pathology with T-cell density and SPARC expression.

Univariate analysis for Pathological features vs. SPARC, FOXP3, CD8 and CD45RO expression from patients with Stage II and III CRC			
Features	Correlation coefficient	n	p value
**SPARC**			
AJCC	−0.02	345	NS
Site, distal vs. proximal	0.03	336	NS
Grade, poor vs. well/moderate	−0.05	258	NS
Vascular invasion, yes vs. no	−0.04	299	NS
Lymphatic invasion, yes vs. no	0.02	198	NS
Perineural invasion, yes vs. no	−0.009	288	NS
Lymphatic response, yes vs. no	0.00	331	NS
Microsatelite instability, yes vs. no	−0.04	330	NS
**FOXP3**			
AJCC	−0.04	329	0.0002
Site, distal vs. proximal	−0.05	321	NS
Grade, poor vs. well/moderate	−0.38	245	0.02
Vascular invasion, yes vs. no	−0.06	286	NS
Lymphatic invasion, yes vs. no	0.24	189	NS
Perineural invasion, yes vs. no	−0.41	275	NS
Lymphatic response, yes vs. no	−0.06	316	NS
Microsatelite instability, yes vs. no	0.11	315	NS
**CD8**			
AJCC	−0.64	316	<0.0001
Site, distal vs. proximal	0.13	310	NS
Grade, poor vs. well/moderate	−0.19	232	NS
Vascular invasion, yes vs. no	0.01	272	NS
Lymphatic invasion, yes vs. no	−0.05	174	NS
Perineural invasion, yes vs. no	−0.69	261	0.01
Lymphatic response, yes vs. no	0.39	303	0.02
Microsatelite instability, yes vs. no	0.70	301	0.0001
**CD45RO**			
AJCC	−0.61	33	<0.0001
Site, distal vs. proximal	0.18	325	NS
Grade, poor vs. well/moderate	−0.14	250	NS
Vascular invasion, yes vs. no	−0.12	289	NS
Lymphatic invasion, yes vs. no	0.10	193	NS
Perineural invasion, yes vs. no	−0.70	278	0.002
Lymphatic response, yes vs. no	0.26	319	0.055
Microsatelite instability, yes vs. no	0.59	318	<0.05

This table illustrates the correlation between different CRC pathological features and T-cell density or SPARC expression using univariate analysis.

Abbreviations: n- number, NS–not significant, AJCC – American Joint Committee on Cancer.

### Adjuvant chemotherapy

TMA collection occurred from 1990-96 and prior to 1994 chemotherapy was not routinely given for CRC. Of the patients in this cohort, 8% of stage II and 44% of stage III patients received adjuvant chemotherapy. SPARC expression and T-cell densities were compared between patients who had, and had not, received adjuvant chemotherapy post-surgical resection (***Table 3***). The ratio of SPARC to vimentin expression was significantly greater in patients with stage III CRC, who had not received adjuvant therapy (p = 0.01), while in patients with stage II CRC, FOXP3^+^ cell density was significantly greater in those patients who had received adjuvant therapy compared to those who had not (p = 0.04).

**Table 3:** Adjuvant chemotherapy.

SPARC, FOXP3, CD8 and CD45RO Expression and adjuvant chemotherapy			
Adjuvant Chemotherapy			
	Yes	No	p value
**Stage II**			
SPARC	0.60	0.53	0.18
FOXP3+	183.38	151.64	0.16
CD45RO	1428.71	1275.43	**0.04**
CD8+	283.12	293.71	0.42
**Stage III**			
SPARC	0.52	0.61	**0.01**
FOXP3+	138.12	131.88	0.38
CD45RO	180.42	179.89	0.49
CD8+	838.96	927.40	0.20

The mean SPARC, FOXP3, CD8 or CD45RO expression levels in patients who did, or did not, receive adjuvant chemotherapy in stage II and III CRC. SPARC was significantly greater in patients with stage III CRC who did not require chemotherapy, whilst FOXP3 was significantly greater in patients with stage II CRC who did receive chemotherapy.

## Discussion

Cell migration or invasion through the basement membrane and distant spread via the lymphatic or vascular systems are inherent characteristics of malignant disease and have a significant impact on disease outcome[3]. These migratory processes require the differential regulation of adhesive and anti-adhesive molecules, and it has been suggested that SPARC may play a key role in the initial processes of tumour invasiveness and ECM modulation is due to its anti-adhesive properties. Treg cells may also contribute to malignant disease by suppressing local anti-tumour immune responses within the tumour microenvironment[29], [30]. The presence of high numbers of FOXP3-positive T cells has been associated with a higher risk of disease recurrence and poor overall survival of patients with solid tumours[29]. AJCC disease staging is based on histological evidence of infiltration and metastasis, it was, therefore, hypothesised that SPARC and FOXP3 expression may be associated with primary CRC disease stage and cancer specific survival.

This study observed a higher ratio of SPARC to vimentin in CRCs, regardless of disease stage, than in control tissue, suggesting a role for SPARC in colorectal carcinogenesis. Stages II, III and IV CRC all had significantly higher SPARC expression than stage I, however, there were no significant differences observed between stages II, III or IV CRC, indicating that although SPARC expression was higher in the intermediate and most advanced stages of CRC, it does not always correlate with the cancer stage as has previously been suggested[6], [18], [31]. These studies, undertaken in human gastric carcinoma[6], CRC[31] breast[18] and oesophageal cancer[32], identified that SPARC was a poor prognosis marker, and was associated with more aggressive and highly metastatic tumours. Our findings, however, demonstrated that higher levels of SPARC expression in the primary CRC was associated with a good disease outcome and a better long-term cancer-free survival >60 months in both stage II and III CRC, and conversely that lower SPARC expression was associated with a more advanced clinical disease stage.

Our findings are consistent with the observation that reduced SPARC levels in primary CRC xenografts can increase tumour resistance to radiation and chemotherapeutic agents[33]. Radiosensitivity and response to 5-fluorouracil and irinotecan in the mouse xenografts, however, was able to be restored by the re-expression of SPARC, while the overexpression of SPARC in CRC xenografts enhanced tumour sensitivity to radiation and chemotherapy[33]. Although prior to 1994 chemotherapy was not routine, in our study, which collected samples from 1990 to 1996, the majority of patients with stage III CRC and higher SPARC expression did not receive chemotherapy. This suggests the possibility that, not only is SPARC capable of sensitising CRC cells to chemotherapy[33], [34], but higher SPARC expression may be associated with other clinical features that the clinician thinks makes the tumour less aggressive and thus not require adjunctive therapy.

SPARC may be potentially anti-tumorigenic through the induction of a microenvironment that is non-permissive to tumour progression[35]. Chlenski *et al*, demonstrated that enhanced SPARC expression in mouse xenografts through transfected cells, resulted in a decrease in smooth muscle actin expression, as well as impaired xenograft growth, inhibition of angiogenesis and more tumour stroma compared to wild-type and vector-control 293 xenografts[35]. Co-culturing of the SPARC-transfected cells with wild-type or vector-control xenografts also resulted in fewer activated fibroblasts that were unable to produce enough ECM to support tumour growth. By altering the composition of the tumour stroma through the inhibition of vascular cells, recruitment of host fibroblasts and prevention of fibroblast activation, it was suggested that SPARC may play a role in “normalising” the tumour stroma, leading to a microenvironment that is not supportive of tumour growth and progression[35].

In concordance with earlier reports that tumour infiltrating FOXP3^+^, CD8^+^ and CD45RO^+^ T cells are associated with better patient survival[8], [10], [28], [36], our study confirms the importance of T cell density as a prognostic marker in CRC. Important findings of this study, however, are that FOXP3 inversely correlated with CRC disease progression and high FOXP3 expression levels specifically correlated with a good disease outcome in stage II CRC. FOXP3 expression was also higher in a good, compared to poor, disease outcome in stage III CRC, but this was not significantly different. A major difference between stage II and III cancers, however, is that stage II cancers are localised to the gut, whilst stage III cancers have lymphatic spread with the tumours taking on a metastatic phenotype within a pro-inflammatory microenvironment. Under these conditions, Tregs fail to inhibit tumour progression and may even contribute to a T-helper (Th)-17 driven pro-carcinogenic process[37]. This may potentially explain why differences between good and poor disease outcome in stage III CRC did not reach statistical significance. FOXP3 was also noted to be associated with low disease recurrence and better long-term survival. This suggests that the levels of FOXP3 expression, as for SPARC, in the patient's primary tumour may play an important prognostic role that could assist in distinguishing between stage II cancers that would, and would not, benefit from post-operative adjuvant chemotherapy. Being able to predict disease outcome from the primary tumour would allow for the development of specific treatment regimes to suit each individual patient.

In most solid tumours including melanoma[38], breast[26], ovarian[39], hepatocellular[40] and pancreatic cancer[41], Tregs have been implicated in tumour progression because of their primary role as a suppressor of cytotoxic T cells and the host's protective anti-tumour inflammatory responses. The work that is presented in this study, however, demonstrates the opposite, where Treg levels are inversely correlated with disease stage and is associated with good disease outcomes. One study that supports our findings postulated that the risk of CRC is greatest when interrelated activities of Treg, intestinal bacteria, and the innate immunity are unable to restore and support systemic homoestasis[37]. The investigators demonstrated that the ability of Tregs to inhibit or suppress cancer was highly dependent upon the type of gut bacteria and IL-10. Individuals with a weakened IL-10 and Treg-mediated inhibitory loop are highly susceptible to the carcinogenic consequences of gut bacteria-induced inflammation and show more frequent inflammation-associated cancers[37]. A functional link between chronic inflammation and cancer has long been suspected, but the crucial molecular pathways that permit communication between cancer cells and inflammatory cell infiltrates largely remain unknown. These concepts are supported by the above study but further functional studies into the role Tregs play in the anti-tumour response are still required in order to explain these observed associations with prognosis and disease recurrence.

In conclusion, this present study confirmed that CD8 and CD45 correlate with CRC disease stage and outcome. It is, however, the first to report on the prognostic significance of SPARC and FOXP3 in distinguishing between good and poor disease outcome in stage II CRC. Furthermore, multivariate analysis demonstrated that FOXP3 had a stronger prognostic value than CD8 and CD45RO, whilst SPARC had the strongest prognostic value of all four markers measured. Although further studies are required before these markers can be employed, the present results suggest that assessment of SPARC and FOXP3 expression in tumour tissue should improve the prognostic classification of stage II and III CRC.
